# Case Report: Bone cement leakage in the right heart: a rare case of misinterpreted echocardiographic findings

**DOI:** 10.3389/fsurg.2025.1608784

**Published:** 2025-07-04

**Authors:** Dongji Kong, Xiaohong Xie, Yong Jin, Yizhen Zhang

**Affiliations:** ^1^ Department of Ultrasonic, Ningbo Yinzhou No.2 Hospital, Ningbo, Zhejiang, China; ^2^ Department of Infection, Ningbo Yinzhou No.2 Hospital, Ningbo, Zhejiang, China

**Keywords:** percutaneous vertebroplasty, bone cement leakage, echocardiography, computed tomography, case report

## Abstract

Bone cement leakage is a relatively common complication following percutaneous vertebroplasty (PVP); however, cement migration to the heart via the venous system is rare, causing severe chest pain and dyspnea. This case reports an 80-year-old male who presented to the Department of Infectious Diseases with mild fever, 2 months after undergoing PVP for a compression fracture of the third lumbar vertebra. A chest computed tomography initially misidentified the lesion as a pacemaker lead within the right ventricle. However, echocardiography revealed that the distal bone cement was embedded in the myocardium of the right ventricular apex, leading to the diagnosis of bone cement leakage. Thereafter, the patient underwent an open-chest procedure with direct cardiac visualization for cement removal in the cardiac surgery department.

## 1 Introduction

Percutaneous vertebroplasty (PVP) is a widely used minimally invasive procedure for managing vertebral compression fractures. It involves injecting bone cement into the compressed vertebra to restore structural integrity and alleviate pain (1). As its use has expanded, reports of complications have increased, most notably bone cement leakage (2). The typical pathway for cement migration into the right ventricle is through the perivertebral venous system, which connects the vertebral venous plexus to the inferior vena cava and, ultimately, to the right atrium and ventricle (3). In severe cases, this can lead to intracardiac and pulmonary artery embolism, posing potentially life-threatening risks. Although cardiac perforation due to bone cement leakage is a recognized complication, several patients with intracardiac cement remain asymptomatic, often diagnosed incidentally, indicating that the potential risk remains underestimated (4). In this case, bone cement leakage was identified in the right ventricle via echocardiography 2 months after PVP. On computed tomography (CT), it was strikingly similar to a pacemaker lead. Due to its distinct shape and relatively stable position, the leaked cement did not cause further cardiac perforation or significant symptoms.

## 2 Case description

**Figure 1:**
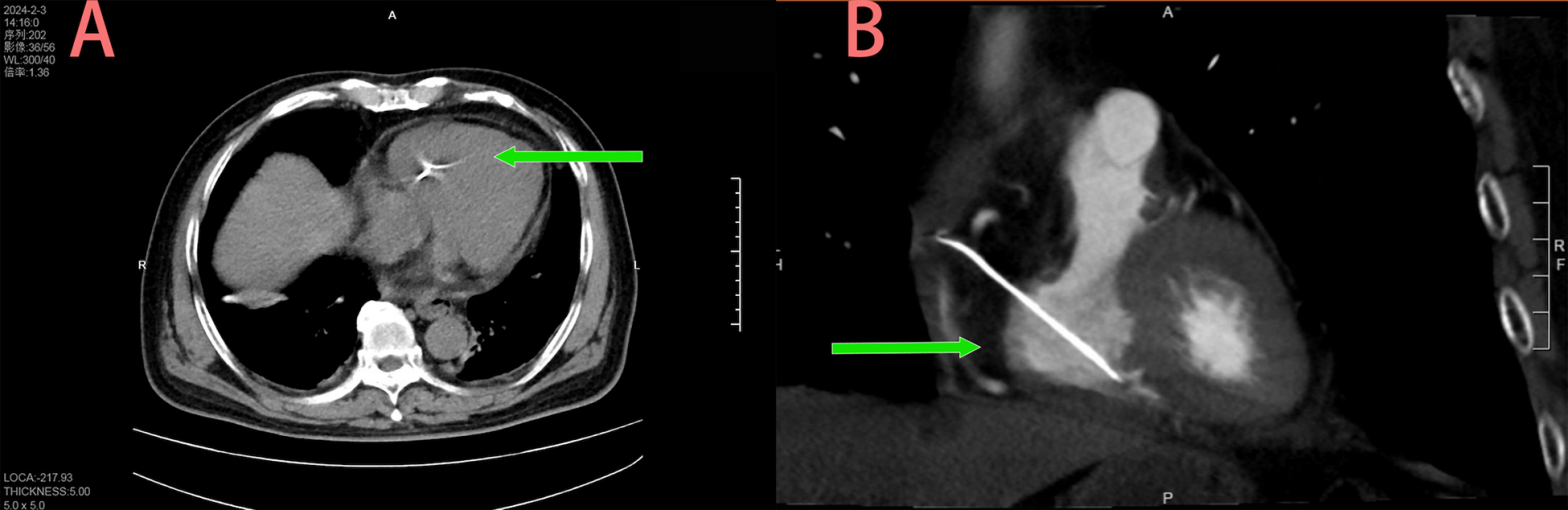
Right intracardiac high density shadow of chest CT **(A)** and reconstruction **(B)**.

**Figure 2:**
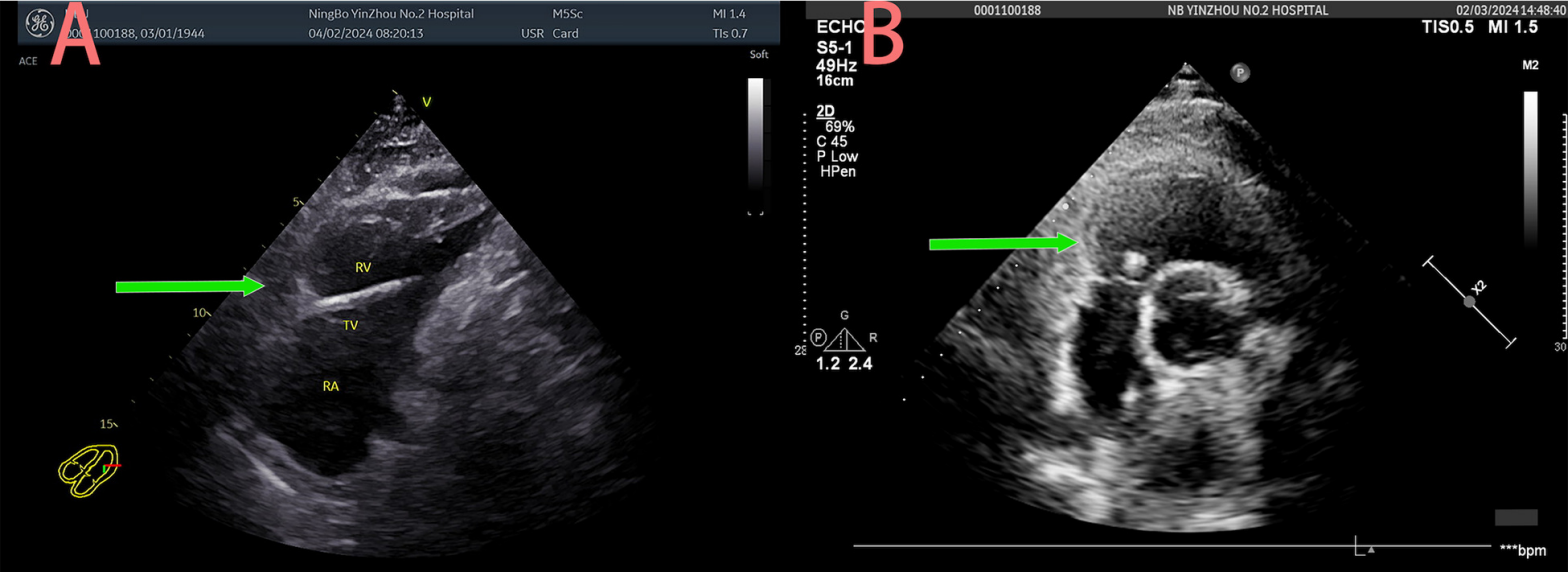
Right ventricle and atrium of hyperechoic foreign body **(A)** right ventricular outflow tract of hyperechoic foreign body **(B)**.

An 80-year-old male was admitted to the Department of Infectious Diseases with a 1-day history of fever and no cardiac symptoms. The patient had no history of hypertension, diabetes mellitus, renal dysfunction, hyperlipidemia, blood transfusion, trauma, smoking, alcohol consumption, or hereditary diseases. A chest CT, performed to investigate suspected respiratory tract infection, revealed a structure resembling a pacemaker lead within the right heart (Figures 1A,B). Electrocardiography showed sinus rhythm, while laboratory tests revealed that cardiac troponin I levels were within the normal limits. In addition, inflammatory markers were abnormally elevated. Transthoracic echocardiography identified an elongated, hyperechoic, rigid foreign body spanning the right ventricle and atrium (Figure 2A). The object exhibited no mobility relative to the heart and had its distal end embedded in the myocardium of the right ventricular apex; hence, no motion or free end was observed. Mild tricuspid regurgitation and pericardial effusion were observed. In the short-axis view of the great arteries, the foreign body extended into the right ventricular outflow tract (Figure 2B). The echocardiographic findings were initially misinterpreted as a pacemaker lead in the right ventricle. The patient was initially diagnosed with a fever of unknown infectious origin. Therefore, initial treatment consisted of intravenous ceftriaxone (2.0 g once daily) for anti-infective therapy in the Department of Infection. After 2 days of treatment, the fever resolved, and inflammatory markers decreased, indicating the effectiveness of the anti-infective therapy. However, further inquiry revealed that the patient had undergone PVP for osteoporotic compression fracture of the third lumbar vertebra 2 months earlier. A multidisciplinary team composed of doctors from the Departments of Infectious Diseases, Ultrasound, and Radiology, confirmed that the patient had no history of pacemaker implantation. Based on the echocardiographic and CT images, intracardiac bone cement leakage was diagnosed. After thorough discussion of the risks associated with the intracardiac foreign body, the patient consented to surgical intervention in the Department of Cardiac Surgery.

**Figure 3:**
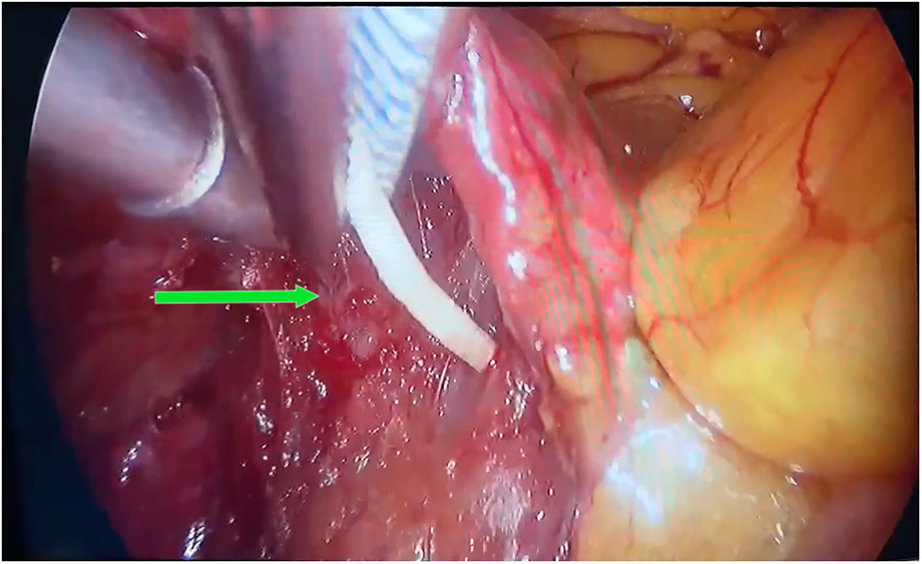
Bone cement visualized in the right atrium.

To prevent further severe complications, the cardiac surgeon proceeded with surgical excision. A 5-cm incision was made at the fourth intercostal space of the right anterolateral chest wall, along with a 1-cm endoscopic port at the sixth intercostal space of the right midaxillary line. Upon opening the pericardium superior to the right phrenic nerve, a rigid, rod-shaped white protrusion of bone cement was observed in the right atrium (Figure 3 and Supplementary Video S1). Cardiac surgeons initially attempted *en bloc* removal of the bone cement using vascular forceps through a minimally invasive approach. Unfortunately, the cement fragmented during the extraction attempts. Subsequently, cardiopulmonary bypass was initiated with induced cardiac arrest, followed by right atriotomy under direct visualization. The tricuspid valve was fully exposed and opened, revealing bone cement located on the right ventricular surface at the junction with the anterior diaphragm, which was subsequently removed (Supplementary Figure S1). Although the procedure proceeded smoothly, the patient's intraoperative blood loss was approximately 1,000 ml. Considering the patient's venerable age and to minimize postoperative complications, he was transferred to the ICU for 2 days of observation and discharged on postoperative day 12 for routine follow-up.

## 3 Discussion

This case involved an 80-year-old male who presented with mild fever and was diagnosed with bone cement leakage into the right ventricle. PVP is a widely used, minimally invasive procedure for treating osteoporotic vertebral compression fractures in the elderly. It primarily involves injecting polymethylmethacrylate into the vertebrae to stabilize the bone and alleviate pain. Bone cement leakage, affecting 30%–65% of patients with osteoporotic vertebral collapse after surgery, is a relatively common complication of PVP (5). While symptoms of leakage vary, severe complications such as pulmonary embolism and cardiac perforation can be fatal (6). Ziad et al. reported that intracardiac bone-cement embolism is a rare complication of PVP or kyphoplasty (7). The bone cement typically enters the right ventricle through the perivertebral venous system, which connects the vertebral venous plexus to the inferior vena cava and ultimately reaches the right atrium and ventricle (3, 8). The incidence of severe complications from bone cement leakage ranges from 2% to 11.5%, emphasizing the importance of early detection and management for patient prognosis (5). Common symptoms of intracardiac embolism are chest pain and dyspnea, which can manifest days, months, or even years after surgery (9). In this case, the patient presented with low-grade fever 2 months after PVP without significant chest pain or dyspnea, offering a rare and unique diagnostic perspective.

In a retrospective study, Zhang et al. performed a polynomial logistic analysis to identify risk factors contributing to segmental venous (S-type) leakage, including sex, cement dispersion type, presence of basal vertebral foramen or fissures, fracture severity, and posterior wall invasion (10). In this study, an 80-year-old male patient presented with a moderate wedge compression fracture of the L3 vertebra and underwent PVP via a bilateral transpedicular approach. The bone cement demonstrated a diffuse distribution pattern, meeting the high-risk criteria defined by Zhang et al. (10). However, these predisposing factors were not adequately identified by the initial clinical team during the perioperative period. This case highlights the critical importance of early identification of patients with high-risk profiles to prevent potential complications. Currently, bone cement leakage is primarily diagnosed through imaging, with CT considered the gold standard (5). In this case, echocardiography combined with CT imaging enabled accurate diagnosis and guided subsequent surgical treatment. Echocardiography offers real-time visualization of bone cement mobility, assessment of cardiac valve structure and function, and facilitates timely repeat examinations. In patients with limited mobility, echocardiography can be performed at the bedside, which is an advantage.

On echocardiography, leaked bone cement may appear as a hyperechoic structure, which may resemble a pacemaker lead, potentially leading to misdiagnosis. The misdiagnosis primarily stemmed from the overlapping echocardiographic characteristics of the two entities, both appearing as hyperechoic linear foreign bodies with similar morphologic features on two-dimensional imaging, potentially obscured by thrombus-induced acoustic interference. This diagnostic challenge was exacerbated by inadequate multiplanar dynamic tracking due to operator inexperience, particularly the failure to systematically assess the fixation of the foreign body to the myocardium using standardized views such as the apical four-chamber and subcostal long-axis projections. Additionally, limited clinical reasoning led to the omission of critical integration of surgical history and multimodality imaging, including cardiac CT angiography. The rarity of the condition and limited clinical awareness among practitioners further compounded the diagnostic dilemma. Key echocardiographic differentiators between intracardiac bone cement emboli and pacemaker leads include anatomic location, imaging characteristics, and pathophysiological features. Bone cement emboli typically lodge deep within the myocardium of the right ventricular inflow tract or free wall, presenting as hyperechoic signals with irregular surfaces and characteristic comet-tail artifacts, with fixed proximal ends that show slight undulation due to blood flow (11). In contrast, pacemaker leads are typically anchored at the apical or septal regions, characterized by smooth spiral configurations, synchronized motion with the cardiac cycle, and intact endothelial coverage (12). Suboptimal echocardiographic resolution and limited clinical experience may increase the likelihood of misinterpretation, thereby delaying accurate diagnosis. Several studies support surgical removal as the preferred treatment for symptomatic intracardiac embolism, given the risks associated with conservative management, including perforation, pericardial tamponade, severe valvular dysfunction, and sudden death (5, 13, 14). This case aims to raise awareness among clinicians about the potential complications of PVP, thereby facilitating early diagnosis, treatment, and prevention of severe outcomes. Overall, while cardiac complications resulting from bone cement leakage are relatively rare, their potential risks should not be underestimated. Echocardiography serves as an effective diagnostic tool, enabling the early detection of bone cement leakage and assessment of its effect on the cardiac function.

## Data Availability

The raw data supporting the conclusions of this article will be made available by the authors, without undue reservation.

## Supplementary material

Supplementary Figure S1 Fragmented bone cement specimen retrieved during surgery.
