# Supplementary figures and images for: Case Report: Bone cement leakage in the right heart: a rare case of misinterpreted echocardiographic findings
Source: Front Surg. 2025 Jul 4;12:1608784. doi: 10.3389/fsurg.2025.1608784 (PMC12271183; doi:10.3389/fsurg.2025.1608784)

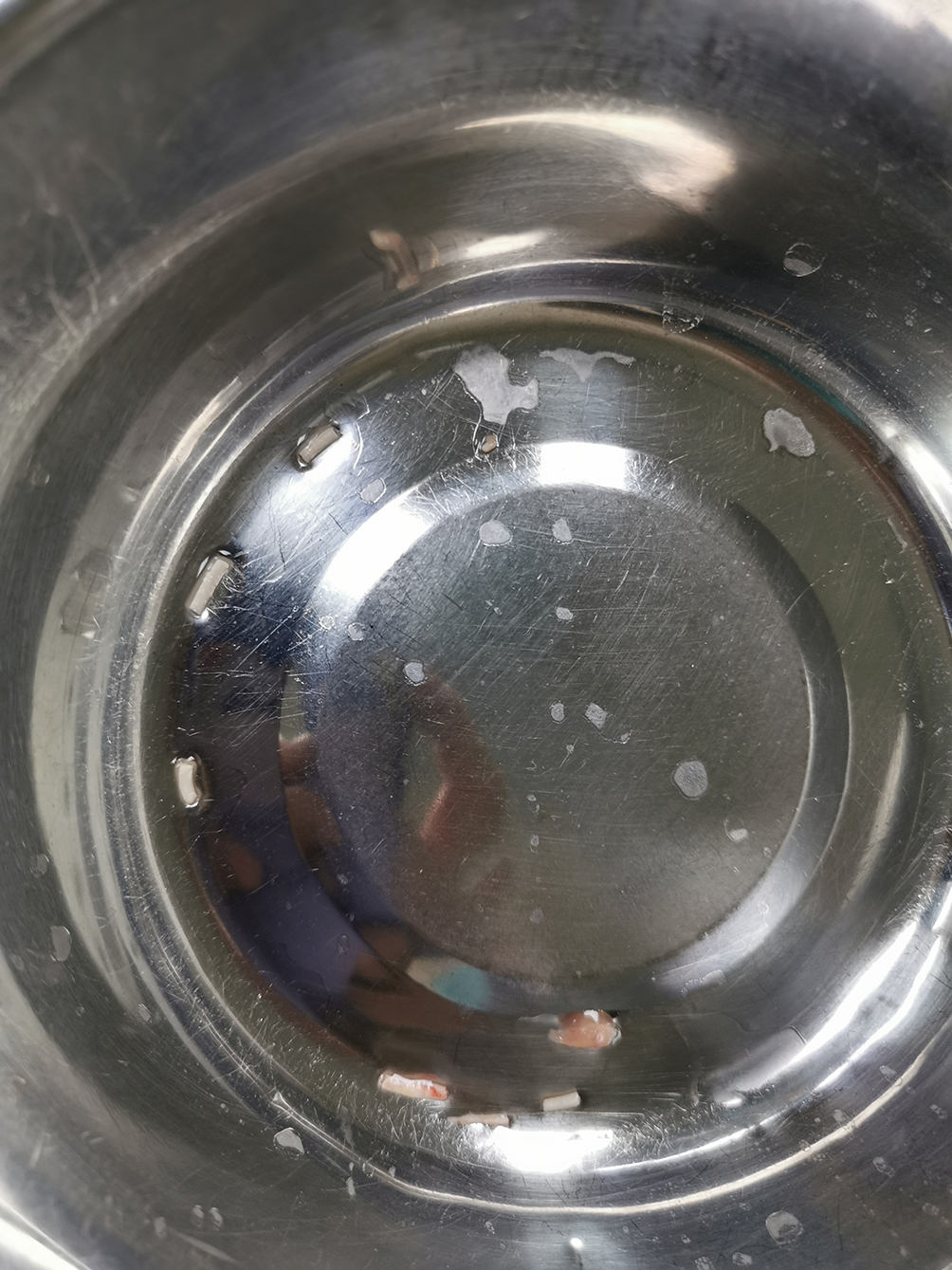

Supplement: Supplementary Figure S1 — Fragmented bone cement specimen retrieved during surgery. [file Image1.tif]
